# Antiulcerogenic Activity and Toxicity of *Bauhinia holophylla* Hydroalcoholic Extract

**DOI:** 10.1155/2015/439506

**Published:** 2015-04-12

**Authors:** A. L. Rozza, D. A. S. Cesar, L. G. Pieroni, L. L. Saldanha, A. L. Dokkedal, F. M. De-Faria, A. R. M. Souza-Brito, W. Vilegas, R. K. Takahira, C. H. Pellizzon

**Affiliations:** ^1^Laboratory of Experimentation of Natural Products, Morphology Department, Institute of Biosciences, Universidade Estadual Paulista (UNESP), 18618-970 Botucatu, SP, Brazil; ^2^Botany Department, Institute of Biosciences, Universidade Estadual Paulista (UNESP), 18618-970 Botucatu, SP, Brazil; ^3^Biological Science Department, Science Faculty, Universidade Estadual Paulista (UNESP), 17033-360 Bauru, SP, Brazil; ^4^Department of Structural and Functional Biology, Institute of Biology, University of Campinas (Unicamp), 13083-862 Campinas, SP, Brazil; ^5^Experimental Campus of the Paulista Coast, Universidade Estadual Paulista (UNESP), 11330-900 São Vicente, SP, Brazil; ^6^Clinics Veterinary Department, School of Veterinary Medicine and Animal Science, Universidade Estadual Paulista (UNESP), 18618-970 Botucatu, SP, Brazil

## Abstract

Several species of *Bauhinia* are used in traditional medicine for the treatment of gastrointestinal diseases, diabetes, and inflammation, among other conditions. The aim of this study was to investigate the antiulcer effect of a hydroalcoholic extract from the leaves of *B. holophylla*. The chemical profile of the extract was determined by HPLC-PAD-ESI-IT-MS. A dose-effect relation was constructed using the ethanol-induced gastric ulcer model in male Wistar rats. Histological analyses and studies of antioxidant and anti-inflammatory activities were performed in stomach samples. The involvement of SH compounds, NO, K^+^_ATP_ channels, and *α*_2_-adrenergic receptors in the gastroprotective effect was evaluated. A toxicity study was performed with a single oral dose of 5000 mg/kg. The extract was composed mainly of cyanoglucoside and flavonol-O-glycosides derivatives of quercetin and myricetin. SH compounds, NO release, K^+^_ATP_ channel activation, and presynaptic *α*_2_-adrenergic receptor stimulation each proved to be involved in the antiulcer effect. The levels of GSH and activity of GR and GPx were increased, and the levels of TNF-*α*, IL-6 and IL-10 were modulated. There was an antidiarrheal effect and there were no signs of toxicity. *B. holophylla* presents antiulcer activity mainly by decreasing oxidative stress and attenuating the inflammatory response, without inducing side effects.

## 1. Introduction

The pathogenesis of gastric ulcer is commonly associated with alterations of physiologic parameters, such as the excessive secretion of gastric acid, disruption of the mucus barrier, formation of reactive oxygen species, lipid peroxidation, and the release of inflammatory mediators, among other factors [1]. Despite the clinical and commercial success of the current treatments for gastric ulcer, there are important clinical needs that remain unmet. Faster and better symptom control, more rapid healing, lack of tolerance, and increased time in the plasma to avoid ineffectiveness, especially at night, are some examples of the persistent goals [2, 3].

The search for a new therapy has driven research to focus on medicinal plants and natural products. In Brazil, several species of* Bauhinia* are known as “pata de vaca” and have been widely used in the treatment of several conditions, such as infections, pain, diabetes, and gastric ulcer [4]. Several ethnopharmacological studies have been carried out on different species of* Bauhinia*. The methanolic and chloroformic extracts of* Bauhinia thonningii* [5] and the chloroformic [6], methanolic [7], and aqueous [8] extracts of* Bauhinia purpurea* previously presented antiulcer effects in rats. Despite the traditional use of the genus* Bauhinia*, including* B. holophylla*, to the best of our knowledge, there are no studies regarding in vivo effects of* B. holophylla*. The present study aimed to investigate the antiulcerogenic activity of the hydroalcoholic extract of* B. holophylla* leaves and the mechanisms underlying its gastroprotective effect.

## 2. Material and Methods

### 2.1. Plant Material and Extraction

Samples of* B. holophylla* leaves were collected in November 2010 at the Botanical Garden of Bauru (22°20′30′′S and 49°00′30′′W), SP, Brazil. Voucher specimens were prepared and identified by Dr. Ângela Maria Studart da Fonseca Vaz and stored at the Herbarium of the Botanical Garden of Rio de Janeiro (Rio de Janeiro/RJ, Brazil) under code number RB 507.043, RB 507.045, RB 507.046, RB 507.47, and RB 507.048. Fresh leaves were dried at 40°C for 48 h. Separated powdered leaves were extracted with EtOH/H_2_O (7 : 3) by percolation at room temperature. The filtrate was concentrated to dryness under reduced pressure at 40°C furnishing the hydroalcoholic extract yielding 29.5% of the dry weight.

### 2.2. Chemical Profile of the 70% EtOH Extract by HPLC-PAD-ESI-IT-MS

The chromatographic profile of the extract of* B. holophylla* was determined on an Accela High Speed LC (Thermo Scientific, San Jose, CA, USA) equipped with a Phenomenex Luna C_18_ (250 × 4.6 mm i.d.; 5 *μ*m) column and a 4 × 3 mm i.d. column guard (Phenomenex), with PAD, and coupled to an Accela (Thermo Scientific) LCQ Fleet with Ion Trap (IT) 3D and ionization by electrospray (ESI). The mobile phase was ultrapure water (eluent A) and methanol (eluent B), both containing 0.1% formic acid. A gradient of 25–100% B over 70 min was applied, with the following conditions: injection volume, 10.0 *μ*L; column temperature, 25°C; and flow ratio, 0.8 mL·min^−1^. UV-vis spectra were recorded between 200 and 600 nm, and the chromatographic profiles were registered at 360 nm. The identification of compounds was carried out by comparison of retention time, maximal UV-vis wavelength, and mass spectral data with those of standards and the literature [9]. The effluent from the HPLC was directed into the ESI probe. The 70% EtOH extract was dissolved in MeOH-H_2_O (8 : 2) and analyzed at a final concentration of 100 *μ*g·mL^−1^.

### 2.3. Animals

Male Wistar rats (200–250 g) from the Central Animal House of UNESP were fed a certified diet with free access to tap water under standard light-dark cycles (12/12 h), humidity (60 ± 1%), and temperature (21 ± 2°C). All rats were fasted for at least 16 hours prior to each experiment because the treatments were orally administered. After each experiment, the rats were euthanized in a presaturated CO_2_ chamber. All efforts were made to minimize the suffering of the rats. All experimental protocols followed the recommendations of the Canadian Council on Animal Care [10] and were approved by the UNESP Institutional Animal Care and Use Committee (permit number 342-CEUA).

### 2.4. Ethanol-Induced Gastric Ulcers: Determination of Dose

Male Wistar rats were distributed into five groups (*n* = 7) and then orally dosed with vehicle (10 mL/kg), carbenoxolone (100 mg/kg), or* B. holophylla* (50, 100, or 150 mg/kg). After 1 hour, the animals received an oral dose of 1 mL of absolute ethanol. One hour after ethanol treatment, the rats were euthanized, and their stomachs were removed [11]. The stomachs were then opened along the greater curvature and washed. The stomachs were scanned, and the ulcer area (mm^2^) was measured using the AVSoft BioView software. After the statistical analysis of the data, the lower effective dose of the 3 doses tested was adopted for all other assays.

#### 2.4.1. Histological Analysis

After scanning, samples from the stomach of each rat were fixed in ALFAC solution (85% alcohol 80, 10% formalin, and 5% acetic acid) and processed in a paraffin tissue processing machine. Sections of the stomach were cut to a thickness of 5 *μ*m and stained with hematoxylin and eosin (HE) or periodic acid-Schiff (PAS) for histological evaluation.

#### 2.4.2. Preparation of Samples for Biochemical Assays

Immediately after the animals were euthanized, the mucosa of each stomach was scraped using two glass slides, homogenized in a phosphate buffer (0.1 M, pH 7.4), and frozen at −80°C until biochemical analysis. The protein concentration of the samples was determined by the method described by Bradford [12].

*(1) Determination of Total Glutathione (GSH) Levels*. The GSH levels in gastric tissue were determined by Ellman's reaction using 5′5′-dithio-bis-2-nitrobenzoic acid (DTNB) [13]. The intensity of the yellow color was read using spectrophotometry at 412 nm.

*(2) Glutathione Peroxidase (GSH-Px) Activity*. The GSH-Px activity was measured by combining 10 mM of reduced glutathione, 4 mM of NADPH, and 1 U enzymatic activity of GSH-Px; the decrease in absorbance was induced by 0.25 mM H_2_O_2_ and monitored every minute for 10 minutes at 365 nm [14].

*(3) Glutathione Reductase (GR) Activity*. The GR activity was measured by monitoring the decrease in absorbance of NADPH induced by oxidized glutathione in phosphate buffer, pH 7.8 at 340 nm. The absorbance was read every minute for 10 minutes [15].

*(4) Superoxide Dismutase Activity (SOD)*. The SOD activity was analyzed by the reduction of nitroblue tetrazolium using a hypoxanthine-xanthine oxidase system (superoxide generation). The absorbance was read every minute for 10 minutes at 560 nm [16].

*(5) Myeloperoxidase (MPO) Activity*. The MPO activity in the gastric mucosa was measured to evaluate the accumulation of neutrophils. The samples were centrifuged at 3,000 ×g for 15 minutes at 4°C. Aliquots of the supernatant were then mixed with a reaction buffer of 50 mM phosphate containing 0.005% H_2_O_2_ and 1.25 mg/mL O-dianisidine dihydrochloride (pH 6.8) and measured at 460 nm [17].

#### 2.4.3. Measurement of the Gastric Mucosal Levels of TNF-*α*, IL-6, and IL-10

The tissue homogenate was centrifuged, and the cytokines were detected in the supernatant using commercial enzyme-linked immunosorbent assay (ELISA) kits for tumor necrosis factor *α* (TNF-*α*) and interleukins 6 (IL-6) and 10 (IL-10) (BioLegend, San Diego, CA, USA).

### 2.5. Involvement of Sulfhydryl (SH) Compounds or Nitric Oxide (NO) in Gastroprotection

The rats were distributed into six groups (*n* = 7). Two groups of rats were intraperitoneally treated with NEM (N-ethylmaleimide 5 mg/kg, an SH compound blocker), two groups were treated with L-NAME (N-nitro-L-arginine methyl ester 70 mg/kg, an NO synthase inhibitor), and two groups were treated with vehicle (10 mL/kg). One hour later, the vehicle or* B. holophylla* 150 mg/kg was orally administered to two groups each. After 60 minutes, all groups were orally treated with 1 mL of absolute ethanol for gastric ulcer induction [18]. Animals were killed one hour after the ethanol administration, and the stomachs were removed, opened along the greater curvature, and scanned; the ulcer area (mm^2^) was determined with the aid of the software AVSoft BioView.

### 2.6. Involvement of K^+^_ATP_ Channels or Presynaptic **α**_2_-Receptors in Gastroprotection

The rats were distributed into six groups (*n* = 7). Two groups of rats were intraperitoneally treated with glibenclamide (5 mg/kg, a K^+^_ATP_ channels blocker), two groups were intraperitoneally treated with yohimbine (3 mg/kg, a *α*_2_-receptors antagonist), and two groups were intraperitoneally treated with vehicle (10 mL/kg). One hour later, the vehicle or* B. holophylla* 150 mg/kg was orally administered to two groups each. After 60 minutes, all groups were orally treated with 1 mL of absolute ethanol for gastric ulcer induction [19]. Animals were killed one hour after the ethanol administration, and the stomachs were removed, opened along the greater curvature, and scanned; the ulcer area (mm^2^) was determined using of the software AVSoft BioView.

### 2.7. Effect of* B. holophylla* on Castor Oil-Induced Diarrhea

Three groups of rats were orally treated with vehicle (10 mL/kg), loperamide hydrochloride (3 mg/kg), or* B. holophylla* (150 mg/kg). After 30 minutes, each rat received 1 mL of castor oil orally. Immediately after ingesting the castor oil, each rat was kept in an individual cage, the floor of which was lined with blotting paper, and the rats were observed for 5 hours. The following parameters were then observed: onset of diarrhea, number of solid, semisolid, and watery feces and total frequency of fecal outputs. Each rat received an evacuation index (EI) expressed according to the formula: EI = 1 × (number of solid stool) + 2 × (number of semisolid stool) + 3 × (number of watery stool) [20].

### 2.8. Effect of* B. holophylla* on Gastrointestinal Motility

Rats were orally treated with vehicle (10 mL/kg), loperamide hydrochloride (4 mg/kg), or* B. holophylla* (150 mg/kg). After 20 minutes, each rat orally received 1 mL of charcoal meal (10% charcoal suspension in 5% aqueous gum arabic). After 30 minutes, rats were euthanized, and the stomach and small intestine were removed. The distance between the charcoal meal and the pylorus was measured and correlated with the distance from the pylorus to the caecum [21].

### 2.9. Acute Toxicity

Male rats (*n* = 10) orally received vehicle or a single acute dose of* B. holophylla* (5000 mg/kg) after 12 hours of fasting. Possible signs and symptoms associated with toxicity were observed at 0, 30, 60, 120, 180, and 240 minutes after the administration and then twice daily for the next 14 days. Body weights were noted daily. At the end of the period, the rats were euthanized, and the kidneys and liver were removed, weighed, and evaluated. Biochemical analyses were performed on the rats' serum to quantify AST (aspartate aminotransferase), ALT (alanine aminotransferase), *γ*-GT (gamma glutamyltransferase), and alkaline phosphatase to evaluate liver damage and creatinine and urea to evaluate kidney damage using the automated biochemical analyzer SBA-200, CELM, Brazil.

### 2.10. Statistical Analysis

We analyzed parametric data using an unpaired *t*-test or a one-way analysis of variance (ANOVA) followed by Dunnett's test and compared the results to the vehicle group or Tukey's test. We performed the analyses using GraphPad InStat or Prism software (GraphPad Software, La Jolla, CA, USA), and the results are presented as the mean ± standard error of the mean (SEM). A value of *P* < 0.05 was considered significant.

## 3. Results

### 3.1. Chemical Fingerprinting

**Figure 1:**
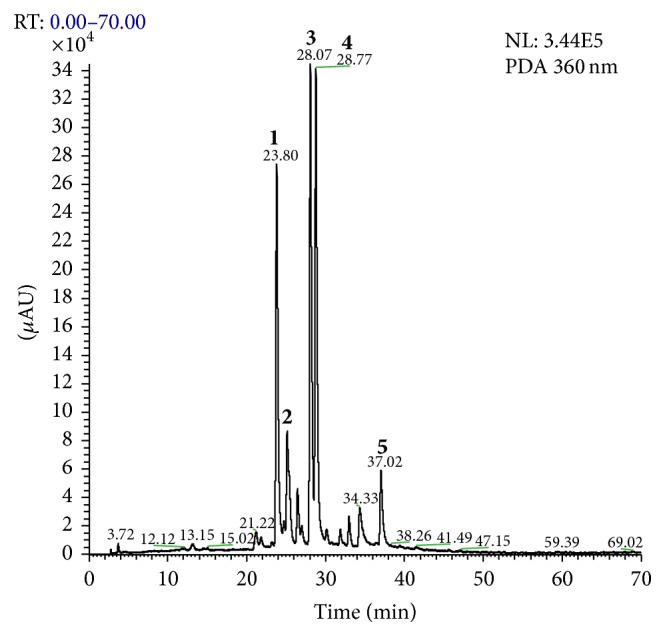
HPLC-PAD analytical chromatogram of 70% EtOH leaf extract of* Bauhinia holophylla*. Experimental conditions: eluents A (H_2_O + 0.1% formic acid) and B (MeOH + 0.1% formic acid). Gradient system: 25–100% B over 70 min. Column: Phenomenex Luna C18 (250 × 4.6 mm i.d., 5 *μ*m). Flow rate: 0.8 mL·min^−1^ and *λ* = 360 nm. Injected volume: 10 *μ*L. Identified compounds: 1, myricetin-O-deoxyhexoside, 2, quercetin-O-hexoside, 3, quercetin-O-pentoside, 4, quercetin-O-deoxyhexoside, and 5, isorhamnetin.

The chemical fingerprinting of the 70% EtOH leaf extract of* B. holophylla* by HPLC-PAD-ESI-IT-MS revealed the presence of flavonol-O-glycosides derivatives of quercetin and myricetin as main constituents (Figure 1).

### 3.2. Ethanol-Induced Gastric Ulcers

#### 3.2.1. Gastric Ulcer Area

**Figure 2:**
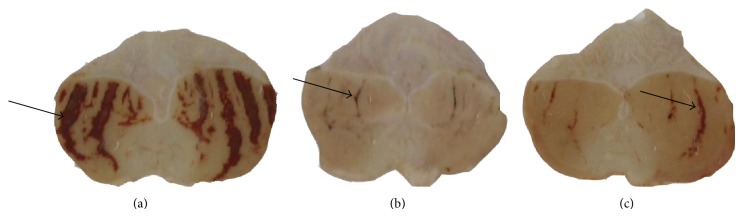
Macroscopic appearance of rat stomachs with ethanol-induced gastric ulcers after treatment with (a) vehicle, (b) carbenoxolone (100 mg/kg), or (c)* Bauhinia holophylla* (150 mg/kg). Arrows indicate ulcer formation.

**Figure 3:**
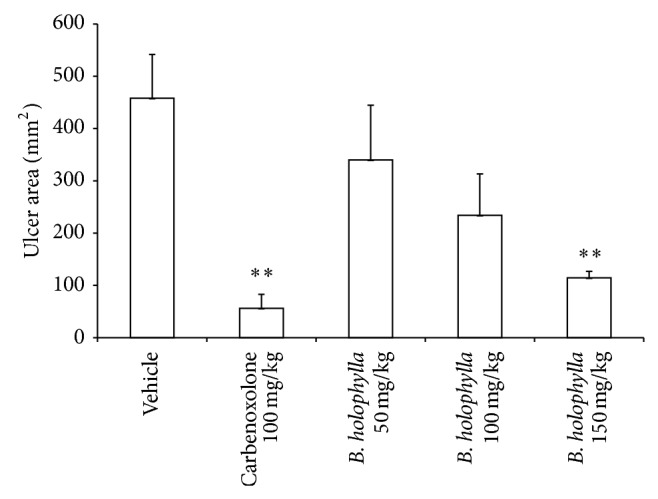
Gastric ulcer area (mm^2^) of rat stomachs with ethanol-induced gastric ulcers after treatment with vehicle, carbenoxolone (100 mg/kg), or* Bauhinia holophylla* (50, 100, or 150 mg/kg). The results are reported as the mean ± SEM. ANOVA followed by Dunnett's test, *P* < 0.05.

The vehicle group presented an average ulcer area of 458.15 ± 83.52 mm^2^, characterized by hemorrhagic bands (Figure 2). The reference drug carbenoxolone provided 88% gastroprotection (*P* < 0.01). Among the groups treated with* B. holophylla* (50, 100, or 150 mg/kg), only the highest dose exhibited a gastroprotective effect (75% gastroprotection, *P* < 0.01), with an average ulcer area of 114.55 ± 12.40 mm^2^ (Figure 3). A dose of 150 mg/kg was used for all subsequent experiments.

#### 3.2.2. Histological Analysis

**Figure 4:**
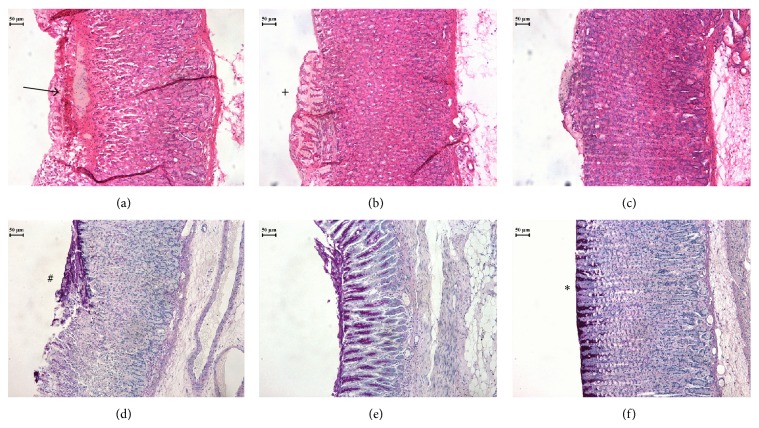
Rat stomachs with ethanol-induced gastric ulcers after treatment with (a, d) vehicle, (b, e) carbenoxolone (100 mg/kg), or (c, f)* Bauhinia holophylla* (150 mg/kg). In the HE staining (a, b, c), the gastric mucosa is more preserved in (b) and (c), besides desquamation, indicated by +. Arrow indicates ulcer formation. In the PAS staining (d, e, f), purple stained area (∗) indicates the mucus secretion in the gastric glands and # indicates glandular damage.

The groups of rats treated with carbenoxolone or* B. holophylla* 150 mg/kg showed a decrease in the occurrence of characteristics typical of ethanol-induced lesions (hemorrhagic lesions, eosinophilic infiltration, desquamation, and glandular damage). The PAS staining provided evidence of the mucus polysaccharides inside the gastric pits of the* B. holophylla*-treated rats. The histological properties of the ulcers are displayed in Figure 4.

#### 3.2.3. Assays of Antioxidant Activity

*B. holophylla* increased the level of GSH (*P* < 0.05) and the activities of the enzymes GSH-Px (*P* < 0.05) and GR (*P* < 0.01). There was a light decrease in the SOD activity; however, this result was not statistically significant. The results of antioxidant activity are presented in Table 1.

#### 3.2.4. Myeloperoxidase (MPO) Activity

The* B. holophylla*-treated rats presented a significant decrease (*P* < 0.05) in the activity of the MPO enzyme in the stomach compared to the vehicle-treated group (Table 1).

#### 3.2.5. Assays of Anti-Inflammatory Activity

*B. holophylla* showed anti-inflammatory activity in the gastric mucosa, as the treatment decreased (*P* < 0.001) the production of the proinflammatory cytokines TNF-*α* and IL-6 and increased the level of the anti-inflammatory cytokine IL-10 (*P* < 0.05) (Table 2).

### 3.3. Involvement of SH Compounds or NO in Gastroprotection

In rats that were pretreated with NEM 5 mg/kg or L-NAME 70 mg/kg, the gastroprotective effect of* B. holophylla* (*P* < 0.01) (150 mg/kg) was reversed, demonstrating the importance of SH compounds and NO production to the gastroprotective effect (Table 3).

### 3.4. Involvement of K^+^_ATP_ Channels or Presynaptic **α**_2_-Receptors in Gastroprotection

The administration of glibenclamide 5 mg/kg (K^+^_ATP_ channels blocker) and yohimbine 3 mg/kg (*α*_2_ presynaptic receptor antagonist) reversed the gastroprotective effect of* B. holophylla* (*P* < 0.01), indicating that both pathways are important to the* B. holophylla* gastroprotection (Table 4).

### 3.5. Effect of* B. holophylla* on Castor Oil-Induced Diarrhea and Gastrointestinal Motility

*B. holophylla* reduced evacuation by 28.73% compared to the vehicle group (*P* < 0.01), indicating an antidiarrheal activity. However,* B. holophylla* treatment showed no interference in the time of transit of the fecal content treated animals compared to vehicle group (Table 5).

### 3.6. Acute Toxicity

There were no signs of toxicity during the period that the rats were observed (14 days). The body weight of the rats progressed normally, and there were no alterations in the biochemical parameters analyzed in the rats' serum, indicating that the dose of 5000 mg/kg did not induce renal or hepatic toxicity with regard to any of the parameters analyzed (Table 6).

## 4. Discussion

This study aimed to investigate the activity of the hydroalcoholic extract of* Bauhinia holophylla* toward ethanol-induced gastric ulcers in rats. Regarding the chemical analysis, derivatives of quercetin and myricetin were revealed in fingerprints of* B. holophylla* extract as the main constituents in accordance with a study of another species of* Bauhinia*,* B. forficata* [9]. In this study, we found that* B. holophylla* only presented a gastroprotective effect in the highest dose (150 mg/kg) tested in ethanol-induced gastric ulcer assay. The stomachs presented hemorrhagic bands parallel to the long axis, and samples of the stomachs were histopathologically analyzed. In contact with the gastric mucosa, ethanol induces several histopathological alterations, such as hyperemia, hemorrhage, inflammatory cell infiltration, and edema [22]. In the HE staining, such alterations are clearly recognized in the vehicle-treated rats. In the group treated with* B. holophylla*, the gastric mucosa appears to be more preserved, with less hemorrhage and inflammatory infiltration. Ethanol ulcerates the gastric mucosa through a direct necrotizing action, which impairs the defensive factors of stomach, such as mucus production. Ethanol also acts by dissolving the mucus barrier that covers the gastric pits [23]. PAS staining colors the mucus barrier in the stomach wall purple; this procedure demonstrated that the mucus layer was preserved in* B. holophylla*-treated rats. These results showed that a possible mechanism for gastric mucosal protection offered by* B. holophylla* is a local mechanism, reinforcing the resistance of the mucosal barrier to the dissolving effect of ethanol, which creates a protective coating that prevents direct contact between ethanol and the epithelium.

Gastric ulcer induction by ethanol is the most prevalent model for studying the pathogenesis of gastric injury and the mechanisms of natural products that exhibit gastroprotection. Ethanol induces ulcers through a variety of mechanisms, including mucus depletion, mucosal excoriation, generation of reactive oxygen species (ROS), and the release of inflammatory mediators. Cells of the stomach have several enzymatic systems that neutralize the harmful activity of ROS. SOD catalyzes the dismutation of the superoxide anion (O_2_^−^) into hydrogen peroxide (H_2_O_2_), which can be metabolized by the activity of GPx and cooperating GR, accompanied by the conversion of glutathione from its reduced form (GSH) into its oxidized form, or by catalase [24]. However, ethanol impairs the activity of SOD, catalase, GSH-Px, and GR and lowers the level of GSH. The reduced antioxidant capacity provoked by ethanol enhances lipid peroxidation, disturbing membrane organization and leading to functional loss and the modification of proteins and nitrogenous bases [25].* B. holophylla* administration prior to ethanol enhanced the activity of GSH-Px and GR and the level of GSH, suggesting an antioxidant activity from* B. holophylla* that prevents the oxidative effects of ethanol.

The administration of absolute ethanol triggers an inflammatory response, which leads to infiltration of inflammatory cells (neutrophils and macrophages) at the site of gastric injury formation [26]. Activated neutrophils release injurious factors, including free radicals, proteolytic enzymes [27], and MPO, which is a marker of neutrophil aggregation and inflammation in the gastric mucosa [28]. MPO activity is increased in the ulcerated stomach [29], as was seen in the vehicle group, but decreased after treatment with* B. holophylla*, providing further evidence for an anti-inflammatory effect of* B. holophylla*. A decrease in MPO activity represents a manifestation of the anti-inflammatory activity [30].

The inflammatory response developed by ethanol administration releases a number of proinflammatory cytokines such as TNF-*α* and IL-6 [28]. TNF-*α* and IL-6 levels are increased in ethanol-induced gastric ulcers, but the harmful effect of these cytokines can be reversed by the administration of natural products with anti-inflammatory activity. We observed that the administration of ethanol leads to an increase in gastric TNF-*α* and IL-6 levels, except in the BH-treated group, remarkably, wherein the production of TNF-*α* and IL-6 was inhibited. The elevated levels of the anti-inflammatory cytokine IL-10 observed in the gastric tissue of rats treated with* B. holophylla* can in part explain the decrease in TNF-*α* production, as IL-10 inhibits the production of TNF-*α* and suppresses the inflammatory response [31]. IL-10 is considered one of the most important anti-inflammatory and immunosuppressive cytokines [32].

The scavenging of free radicals generated by lesive agents and the recycling of antioxidants are also performed by SH compounds [33]. SH compounds are responsible for maintaining disulfide bridges, which in turn maintain the protective barrier of mucus cohesive. The generation of ethanol-induced gastric ulcers is associated with a decrease in SH compounds [34], but the treatment with* B. holophylla* was able to circumvent this process.

NO release from endothelial cells maintains gastric mucosal integrity through interacting with neuropeptides, decreasing lipid peroxidation, and modulation of hydrochloric acid and bicarbonate and mucus secretion [35]. Oral administration of ethanol leads to an increase in NO production, through induction of iNOS (inducible NO synthase) expression [36]. NO increases cyclic guanosine monophosphate levels, leading to activation of the ATP sensitive potassium channels (K^+^_ATP_), in a sequence of events that ends in gastroprotection [37]. K^+^_ATP_ channels mediate gastroprotection through the stimulation of gastric microcirculation and the inhibition of neutrophil activation and subsequent superoxide production [38]. Blocking NO production or K^+^_ATP_ channels reversed the gastroprotection offered by* B. holophylla*. NO release and subsequent K^+^_ATP_ channel activation are a pathway that contributes to the gastroprotective mechanism of BH.

Gyires et al. [39] concluded that peripheral *α*_2_-adrenoceptors are involved in gastric mucosal protection. Rats that had the presynaptic *α*_2_-adrenoceptors antagonized by yohimbine presented a decrease in the gastroprotection, even in vehicle-treated or* B. holophylla*-treated groups, indicating the participation of these receptors in the gastroprotection offered by* B. holophylla*.

Antidiarrheal activity is an advantage for the antiulcer agents because relaxation of circular muscles and flattening of the folds in the stomach lead to an increase in the mucosal area exposed to the necrotizing agent, decreasing the incidence of ulcers [40]. Treatment of diarrhea can be performed through decreasing peristalsis or decreasing secretion from the intestinal mucosa [41].* B. holophylla* offered antidiarrheal activity through decreasing the incidence of watery stools, possibly through reabsorption of water from the intestinal content, because this effect was not accompanied by a decrease in the gastrointestinal motility. Diarrheal disease is the second leading cause of death in children under five years old and is responsible for killing approximately 760,000 children every year [42].

The acute toxicity study of* B. holophylla* showed the nontoxic nature of the extract at a dose of 5000 mg/kg. During the subsequent 14 days, the animals behaved normally, including their consumption of water and food. The enzymes ALT and AST are measured to indicate liver dysfunction; in this case, their production increased [43]. In the same way, high levels of urea and creatinine are indicators of nephrotoxicity [44]. Analysis of the rats' serum did not identify alterations in these parameters, suggesting that oral treatment with* B. holophylla* is safe when administered acutely.

In conclusion, the gastroprotective effect of* B. holophylla* involves the maintenance of mucus barrier through the participation of SH compounds, NO release, K^+^_ATP_ channel activation, presynaptic *α*_2_ receptor stimulation, and antioxidant and anti-inflammatory activities, as well as an antidiarrheal effect and the absence of acute toxicity.

## Figures and Tables

**Table 1:** Effect of *Bauhinia holophylla* (150 mg/kg) on the levels of MPO (myeloperoxidase), GSH (glutathione), GSH-Px (glutathione peroxidase), GR (glutathione reductase), SOD (superoxide dismutase), and MPO (myeloperoxidase) in rat stomachs after ethanol-induced gastric ulcers.

	Vehicle	Carbenoxolone	*Bauhinia holophylla *
GSH	25.88 ± 2.67	54.48 ± 6.78^∗∗^	42.82 ± 1.69^∗^
GSH-Px	96.07 ± 7.21	108.30 ± 14.67	136.80 ± 8.42^∗^
GR	31.51 ± 1.93	40.95 ± 3.12^∗^	42.50 ± 1.00^∗∗^
SOD	3.33 ± 0.50	1.65 ± 0.38^∗^	2.60 ± 0.48
MPO	0.07 ± 0.00	0.04 ± 0.00^∗∗^	0.05 ± 0.00^∗^

GSH level is expressed in nmol/mg of protein; GPx and GR are expressed in nmol/min/mg of protein; SOD and MPO are expressed in U/mg of protein. The results are reported as the mean ± SEM. ANOVA followed by Dunnett's test, ^∗^ *P* < 0.05, ^∗∗^ *P* < 0.01.

**Table 2:** Effect of *Bauhinia holophylla* (150 mg/kg) on the levels of TNF-*α*, IL-6, and IL-10 cytokines in rat stomachs after ethanol-induced gastric ulcers.

	Vehicle	Carbenoxolone	*Bauhinia holophylla *
TNF-*α*	2076.00 ± 122.80	1307.00 ± 283.30^∗^	11.69 ± 5.04^∗∗∗^
IL-6	1273.00 ± 57.56	854.60 ± 169.30^∗^	80.29 ± 23.99^∗∗∗^
IL-10	1923.10 ± 98.15	4607.00 ± 502.50^∗∗∗^	3317.52 ± 399.20^∗^

The results are expressed as pg/mg of protein and reported as the mean ± SEM. ANOVA followed by Dunnett's test, ^∗^ *P* < 0.05, and ^∗∗∗^ *P* < 0.001.

**Table 3:** Effect of *Bauhinia holophylla* (150 mg/kg) on ethanol-induced gastric ulcer area (mm^2^) in rats that were pretreated with L-NAME (NO synthase inhibitor) or NEM (SH compound blocker).

Pretreatment (i.p.)	Treatment (p.o.)	Ulcer area (mm^2^)	Gastroprotection (%)
Vehicle	Vehicle	643.71 ± 113.76	—
	*B*. *holophylla *	220.42 ± 68.67^∗∗^	65.75

L-NAME 70 mg/kg	Vehicle	617.74 ± 116.73	—
	*B*. *holophylla *	419.24 ± 131.32	32.13

NEM 5 mg/kg	Vehicle	460.38 ± 136.06	—
	*B*. *holophylla *	608.93 ± 215.24	—

The results are reported as the mean ± SEM. Unpaired *t*-test, ^∗∗^ *P* < 0.01.

**Table 4:** Effect of *Bauhinia holophylla* (150 mg/kg) on ethanol-induced gastric ulcer area (mm^2^) in rats that were pretreated with glibenclamide (K^+^_ATP_ channels blocker) or yohimbine (presynaptic *α*_2_ adrenoceptors antagonist).

Pretreatment (i.p.)	Treatment (p.o.)	Ulcer area (mm^2^)	Gastroprotection (%)
Vehicle	Vehicle	511.51 ± 103.42	—
	*B. holophylla *	159.52 ± 36.66^∗∗^	68.81

Glibenclamide 5 mg/kg	Vehicle	501.18 ± 258.95	—
	*B. holophylla *	306.47 ± 83.28	38.85

Yohimbine 3 mg/kg	Vehicle	829.40 ± 264.30	—
	*B. holophylla *	422.62 ± 174.13	49.04

The results are reported as the mean ± SEM. Unpaired *t*-test, ^∗∗^ *P* < 0.01.

**Table 5:** Evacuation index and intestinal motility (cm^2^) after oral treatment with vehicle, loperamide hydrochloride, or *Bauhinia holophylla* (150 mg/kg).

	Vehicle	Loperamide	*B. holophylla *
Evacuation index	13.33 ± 0.21	7.16 ± 0.16^∗∗^	9.50 ± 0.22^∗∗^
% of evacuation inhibition	—	46.28	28.73
Intestinal motility	40.05 ± 2.94	17.66 ± 2.33^∗^	40.24 ± 3.91
% of motility inhibition	—	55.90	—

The results are reported as the mean ± SEM. ANOVA followed by Dunnett's test, ^∗^ *P* < 0.05 or ^∗∗^ *P* < 0.01.

**Table 6:** Effect of orally administered *Bauhinia holophylla* (5000 mg/kg) on rats' body weight and serum biochemical parameters.

	Vehicle	*Bauhinia holophylla *
Initial body weight (day 0)	196.70 ± 5.31	203.08 ± 4.73
Final body weight (day 14)	290.60 ± 8.29	298.45 ± 8.19
AST	82.00 ± 4.71	77.71 ± 2.22
ALT	43.42 ± 2.17	47.00 ± 2.70
Gama-GT	6.45 ± 0.58	5.65 ± 0.90
Alkaline phosphatase	271.57 ± 21.51	252.57 ± 26.51
Creatinine	0.30 ± 0.02	0.28 ± 0.02
Urea	51.71 ± 2.25	50.57 ± 1.11

Body weights are expressed in g. AST, ALT, *γ*-GT, and alkaline phosphatase are expressed in U/L; creatinine and urea are expressed in mg/dL. The results are reported as the mean ± SEM. Unpaired *t*-test.
